# Silver release from dentine treated with combinations of silver diamine fluoride, potassium iodide and etching

**DOI:** 10.1080/26415275.2023.2191634

**Published:** 2023-04-20

**Authors:** Frode Staxrud, Rune Becher, Morten Syverud, Naomi Azulay, Håkon Valen

**Affiliations:** Nordic Institute of Dental Materials, NIOM, Oslo, Norway

**Keywords:** Silver diamine fluoride, caries arrest, silver release

## Abstract

For individuals with very high to extremely high caries activity and poor control of daily oral hygiene, a simple treatment for arresting their caries activity is necessary. Silver Diamine Fluoride (SDF) has become increasingly common for this purpose due to its efficacy and ease of application. To avoid or reduce tooth discoloration after SDF treatment potassium iodide (KI) may be applied. However, the release of silver from SDF-treated tooth surfaces may be of concern. Thus, the aim of the present study was to quantify the amount of silver leached in both a short- and long-term perspective. In this *in vitro* experiment we measured the cumulative release of silver from SDF-treated dentin surfaces with and without imminent application of KI, and with and without phosphoric acid etching as pre-treatment, after 24 h and weekly for four weeks. The release of silver was highest after 24 h for all treatment groups, with a significant drop after this point. When etching was not used, the use of KI did not affect the release of silver. However, when etching was used, there was a significantly lower silver release when KI was also used compared to when KI was not used. This effect was largest for the first two weeks, after which the difference was smaller as all groups released low amounts of silver.

## Introduction

For patients suffering from very high to extremely high caries activity, semi-permanent methods to arrest the caries process may be necessary. These patients may be individuals with cognitive and motoric impairments, in nursing homes or at home, or young patients that have difficulties coping with regular dental treatment due to anxiety or immaturity. Early Childhood Caries (ECC) is a serious problem for many children and their families. Children with heavy caries burdens and insufficient daily care, maybe without access to dental care, need to be taken care of to gain control of their caries problem [1–3].

Today Silver Diamine Fluoride (SDF) is used for the purpose of caries arrest [4,5] and treatment of dentin hypersensitivity [6]. Fluoride-metal compounds like SDF has shown cariostatic effect when used on active carious lesions [3,7–9]. The use of SDF has become increasingly popular in the endeavor to help children and frail elderly conquer their caries problems [5,10]. SDF is an inexpensive and easy-to-use topical medicament used extensively in many countries. However, discoloration of dentine is an aesthetic challenge when using SDF, but this can largely or at least to some degree be avoided by the use of potassium iodide (KI) [11,12].

Due to potential toxicity of silver (Ag), its amount released from SDF treated dentine surfaces needs to be quantified for reasons of safety especially when used on children or elderly. In a report from WHO on silver in drinking water it was stated that considerable uncertainties remain regarding the toxicity of silver (ionic silver and silver nanoparticles). Thus, the data were inappropriate for deriving a formal guideline value (WHO/HEP/ECH/WSH/2021.7). However, Hadrup and Lam suggested 2.5 µg/kg BW/day as a Tolerable Daily Intake (TDI) of silver [13]. Although the volume of applicate SDF is extremely low, the concentration of silver is high (as shown in Table 1). Thus, we consider it relevant to elucidate whether the concentrations of released silver confer the possibility of toxicity.

**Table 1.** The SDF material used in this experiment was Riva Star, SDI, Australia, which comes in two bottles.

Riva Star, SDI, Australia	Content	Concentration	LOT number 11676961
Bottle 1	Silver Diamine Fluoride (SDF: AgF(NH_3_)_2_)		210334
		Silver fluoride (35–40% weight)	
		Fluoride (5–6%, 44 800 ppm)	
		Silver (25%, 253 870 ppm)	
		Ammonia (15–20% weight)	
Bottle 2	Potassium iodide (KI)		210273

Potassium iodide, KI, is determined not to be hazardous according to available Safety Data Sheet. Total pH 8-9 for the solution.

The aim of the present *in vitro* study was to quantify passive Ag release from dentine surfaces, treated with a commercially available product containing silver diamine fluoride (SDF) with and without imminent application of potassium iodide (KI), and with and without phosphoric acid etching (37%) as pre-treatment, both after the first 24 h and then weekly the next 4 weeks. These values were compared to known toxicological values available in literature.

## Materials and methods

Silver release test was generally performed according to ISO 10271:2011 Dentistry – ‘Corrosion test methods for metallic materials’ [14] referred to under ISO 22674:2016 ‘Dentistry – Metallic materials for fixed and removable restorations and appliances [15]’. However, the test was modified for the present research project as described below. The purpose of the choice of method was to investigate maximum release of silver passively released into water from dentine during the first 28 days. We did not want to investigate how proteins in saliva may bind the released silver.

Test substrates were produced by using human 3^rd^ molars (< 6 months post extraction). Extracted non-restored human molars were used, taken from a biobank at NIOM with permission to be used for adhesive testing (2013/413 and 2014/457 approved by Regional Committees for Medical and Health Research Ethics, Norway). The teeth were not sterilized. They were cut horizontally in 2 mm thick discs exposing dentine. All organic material (pulp tissue) was eliminated. They were ground with silicon paper, FEPA#500 at the side determined for SDF treatment. To avoid dehydration of the substrates, they were always kept at 100% air humidity.

Dentine surfaces were masked with plastic tape (insulation tape) with a circular aperture, diam. 3 mm, for control of treated area. Phosphoric acid gel was applied on the teeth in the etching pre-treatment groups (group 3 and 4) and rinsed off after 10 s with water. Subsequently, the surface was air dried before application of SDF.

Regarding exposure time and rinsing of SDF, we used the method recommended by the manufacturer. This implies application of SDF by swabbing the actual surface with a micro brush and immediate blot dry the swabbed surface with tissue paper. When KI was used, it was applied on wet SDF before blot drying.

All substrates were treated with SDF, Riva Star, SDI (Australia). Material used is presented in Table 1. An overview of the test groups and pre-treatments are given in Table 2.

**Table 2.** Different applications and pre-treatments for release of silver test. N = number of test specimens.

Treatment groups		*n*	Pre-treatments
1	SDF	12	None
2	SDF + KI,	12	None
3	Etch + SDF	12	Etch (pre-treatment with 37% phosphoric acid, 10 sec + water spray + air drying)
4	Etch + SDF + KI	12	Etch (as group 3)
5	Control 1	1	Dentine slab without any application (negative control tooth)
6	Control 2	1	MilliQ water only (negative control water)

After the different treatments of dentine as specified in Table 2, and according to manufacturer’s manual, specimens were kept in 5 ml MilliQ water allowing silver to passively leach into the water. All MilliQ water was sampled after each time period (24 h, 7 days, 14 days, 21 days and 28 days), and replaced with 5 ml new MilliQ water each time i.e. first time after 24h and then every week. Sampled water was kept in tight plastic vials. The analysis of the amount of silver release was performed by ICP-MS (Inductively Coupled Plasma Mass Spectroscopy) by Sheffield Analytics, Sheffield, UK, with detection limit of 1.0 µg/L as reported by Sheffield Analytics. All samples were diluted at least 1:2, dilution for some samples were 1:10 or 1:50 if the concentration was high. Some results were double checked with ICP-OES (Inductively Coupled Plasma Optical Emission Spectroscopy) to secure quality, as reported by Sheffield Analytics.

### Statistical analyses

A two-way ANOVA model with the groups, time-points and their interaction as explanatory variables was used to compare the short- and long-term release of silver with contrast analysis. In addition, the difference between the groups at 24 h was compared with Tukey tests.

Another ANOVA model was fitted to inspect possible interactions between etching, KI, and time of treatment. Etching and KI were modelled as dichotomous variables, and time as a categorical variable. The 24-h time point was excluded due to the big difference in standard deviations (heteroscedasticity) compared to the other time points. All possible interactions, including the three-way interaction of the independent variables, were included in the model. The different time points were compared with Tukey tests.

All analyses were done in R (version 4.2.1). The code is available from the authors on request.

## Results

The total silver release for each treatment group at all time-points are presented in Table 3. Tukey tests showed that the total silver release after 24 h was significantly lower when dentine was etched before application with SDF in combination with KI compared to the other application protocols (*p* < .05 for all comparisons). Contrast analysis showed a significant decrease in silver release after 24 h (*p* < .001).

**Table 3.** The table shows release of silver after 24h and 1 to 4 weeks, measured in µg/cm^2^ from SDF treated dentine surfaces.

		Release of silver			
Mean µg/cm² (SD)	24h	1 week	2 weeks	3 weeks	4 weeks
SDF (*n* = 11)^a^	97.3 (39.6)	5.2 (8.9)	1.8 (3.5)	2.6 (2.0)	1.6 (1.3)
SDF + KI (*n* = 12)	114.2 (190.4)	7.5 (12.2)	1.3 (2.5)	0.8 (1.4)	0.8 (0.3)
Etch + SDF (*n* = 12)	133.7 (125.6)	19.3 (17.0)	17.9 (11.6)	5.4 (3.9)	3.8 (2.4)
Etch + SDF + KI (*n* = 12)	8.1 (15.0)	1.2 (1.9)	0.4 (0.8)	0.4 (0.6)	0.2 (0.8)

The reduced release of silver by time after 24h, shown in the table, is statistically significant (*p* < . 001 from contrast analysis). ^a^The SDF group contain only 11 specimens as one outlier was removed. The high SD values may be explained by the fact that the substrate is dentine, which is an organic variable anisotropic structure. The concentration unit given from Sheffield analytics was mg/L. This is converted to µg/cm^2^ for easier interpretation regarding application area in clinic.

There was a significant three-way interaction between the dichotomous KI and etching variables and time (*p* = .001). A clear effect of KI was observed during the two first weeks when etching was used as pre-treatment, after which the difference became smaller. Tukey tests showed a significant difference in silver release between weeks 1 and 3 (*p* < .001), 1 and 4 (*p* < .001), and 2 and 4 (*p* = .033).

The concentration unit given from Sheffield analytics was mg/L. We have converted this to µg/cm^2^ because it is easier to relate to a relevant size of tooth application area in clinic. The treated area of dentine was 0.07cm^2^ and the silver leaked out in 5 ml of water. The formula used for the relevant conversion is µg/cm^2^=mg/L * (0.005 L * 0.07065 cm^2^) * 1000.

## Discussion

**Figure 1.**
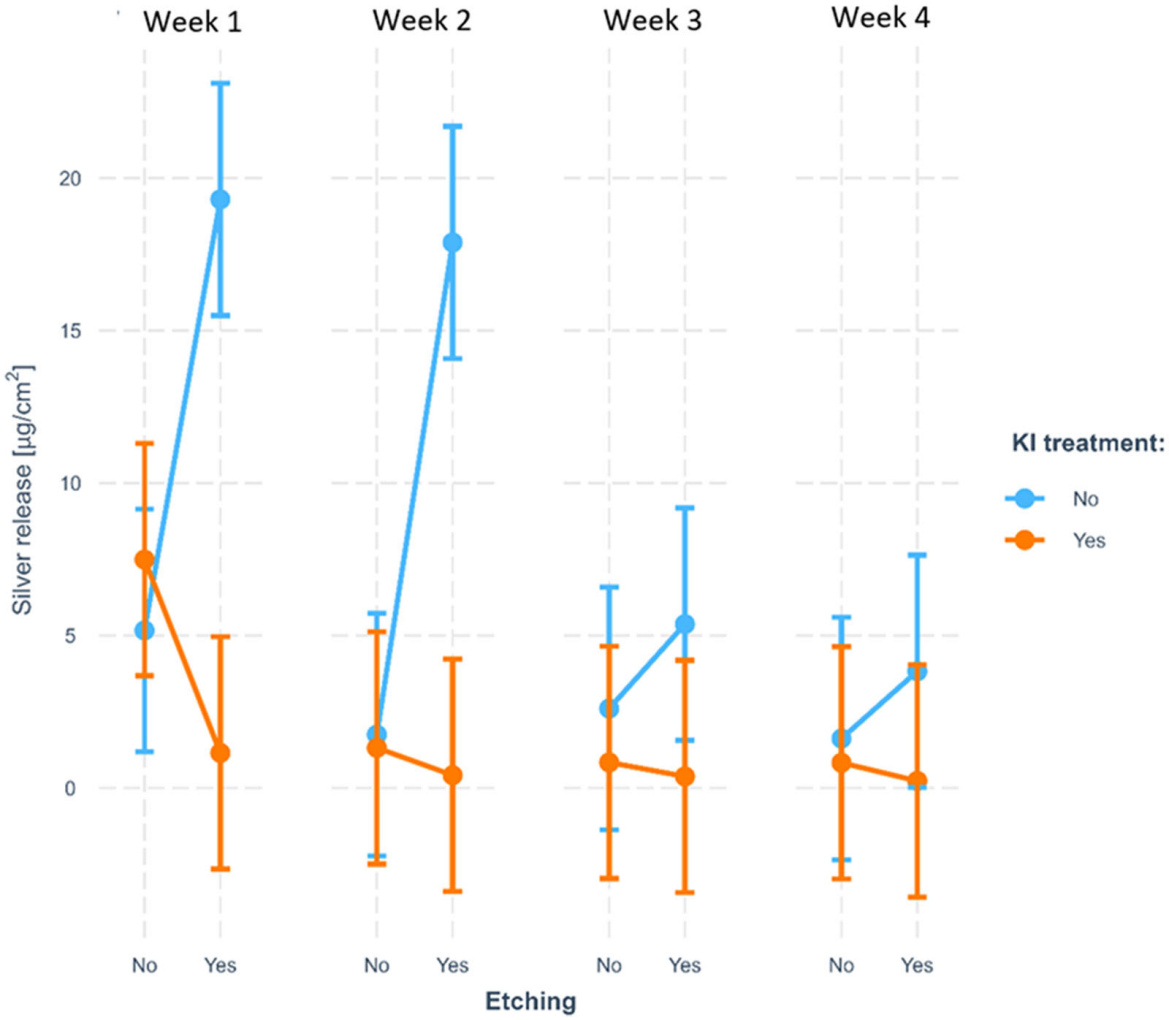
The interaction between etching, KI, and time: The interaction between etching and KI is different at the different time points. We see a clear effect of KI when etching is used during the two first weeks, after which the difference becomes less visible. The error bars represent 95% confidence intervals.

The combination of etching the dentine surface with phosphoric acid before application of SDF and application of KI directly on wet SDF afterwards, significantly reduced silver release after 24 h compared to the other application protocols (Table 3). The three-way interaction between etching, KI and time was statistically significant in the ANOVA model, meaning that the effects of etching and KI differs over time. This is presented graphically in Figure 1 as the two-way interaction between KI and etching at each time point. From this figure we can conclude that the interaction of KI and etching is strong at the two first time points (the amount of silver released when etching is used as a pre-treatment highly depends on the use of KI), while it is negligible at the two latest time points (the amounts are more similar).

SDF has been approved by the Food and Drug Administration (FDA, USA) for treatment of hypersensitivity. In addition SDF is used off-label for treatment of carious lesions [3]. Recently, the World Health Organization (WHO) recommended the use of SDF for caries prevention and treatment as part of a Minimal Intervention strategy [10].

Application of SDF is simple and uncomplicated compared to other operative treatment options and can be performed anywhere without advanced equipment. Isolation of actual lesion is easily handled, and application with micro brush before immediate blot drying suffice. Low treatment cost per application may make SDF application a cost effective treatment for caries arrest [16]. The major criticism against SDF is discoloration of caries lesions into brown/black appearance. When applying potassium iodide (KI) immediately on wet SDF, a white silver-iodide precipitate is created. This largely prevents the discoloration to begin with, but arrested carious lesions have a tendency to darken by time anyway [11].

When etching was not used, the use of KI did not affect the release of silver. However, when etching was used in combination with KI, there was a significantly lower silver release. This effect was largest for the first two weeks, after which the difference was smaller as all groups released low amounts of silver. A possible explanation is that when only etching and SDF is applied, the surface area will be increased but as KI is not present to bind silver the result will be higher release of silver. When etching is not performed, the surface has normally a smear layer that prevents deposition of SDF and KI and possibly AgI. Besides, there is much smaller surface area for the different ions involved to adhere to. We would hypothesize that applications of KI create an AgI precipitate on the surface, which has low water solubility. Subsequently, less silver is released.

Based on animal studies, Haderup and Lam proposed a Tolerable Daily Intake (TDI) for silver of 2.5 µg Ag/kg BW/day [13],. This TDI was based on increased levels of cytokines in plasma of mice orally administered silver nano particles. The authors reported a NOAEL (No Observed Adverse Effect Level) of 0.25 mg Ag/kg BW per day. The authors applied an uncertainty factor of 100, thereby reaching their proposed TDI level.

Assume that a child receives SDF on 1 cm^2^ for caries arresting treatment. The highest immediate silver release in our study was observed to be 511 µg pr. cm^2^, (SDF + KI). There is not reported any kind of acute toxicity for these levels of silver [17,18]. Thus, in a worst-case scenario for a child of approximately 3 years and a body weight of 15 kg, the suggested TDI of Hadrup and Lam would be exceeded after the first 24 h for an area of 1 cm^2^. However, if we assume a linear trend in silver release after the first 24 h, the TDI would not be exceeded after this.

Typical patients considered relevant for SDI treatment include children heavily burdened with early childhood caries, reluctant to conventional chairside dentistry, frail elders, often medically compromised or unable to attend dentist. Noteworthy, it is a minimally invasive and inexpensive treatment. The findings in the present study suggests that it should be safe to use SDF within a margin of safety, with or without KI, even in children. Older patients in hospital, nursing homes or home care should be at least similarly safe when SDF with KI is used for reasons of caries treatment.

The clinical benefit for use of SDF is mainly caries arrest, pain reduction, and possibly to postpone extraction of deciduous molars severely deteriorated by caries. The importance of keeping these temporary molars as placeholders until eruption of permanent premolars is undebatable. An SDF-treated tooth (with KI) which is etched as pre-treatment could easily be sealed with glass-ionomer restorative material (as proposed by the manufacturer, SDI), and serve the patient for this purpose. If the seal is not complete over time, the SDF underneath would possibly be able to stop caries advance. Use of SDF is mainly an atraumatic treatment with little or no use of burs. Children are not exposed to anesthetics or drilling with burs, and hopefully they do not develop anxiety for dentists or dental treatment.

The question of adverse effects needs a few comments. Saforide™ (Toyo Seiyaku Kasei Co. Ltd., Osaka, JP) has been used in Japan for more than 80 years. Despite the presumed toxicity of silver, not one single adverse event has been reported to the Japanese authorities [3]. The manufacturer has sold more than 2 million multi-use containers. Only 3 cases of mild, white, reversible changes on adjacent mucosa have been reported, which would be anticipated due to the high pH (pH 8-9) of the amine-group. No adverse pulpal response has been observed. Silver allergy is however a contraindication.

A main limitation of our study is that its results are confined to release of silver from treated dentine slabs only.

## Conclusion

*In vitro* silver release after application of SDF was highest during the first 24 h. When etching was not used, KI did not affect the release of silver. However, when etching was used in combination with KI, a significantly lower silver release was observed. This effect was most pronounced for the first two weeks, after which the difference became smaller as all groups released low amounts of silver.
